# Efficacy of the motile sperm organelle morphology examination (MSOME) in predicting pregnancy after intrauterine insemination

**DOI:** 10.1186/1477-7827-9-120

**Published:** 2011-08-23

**Authors:** Livia D Akl, Joao Batista A Oliveira, Claudia G Petersen, Ana L Mauri, Liliane FI Silva, Fabiana C Massaro, Ricardo LR Baruffi, Mario Cavagna, Jose G Franco

**Affiliations:** 1 Department of Gynaecology and Obstetrics, Botucatu Medical School, São Paulo State University - UNESP, Botucatu, Brazil; 2 Center for Human Reproduction Prof. Franco Jr., Ribeirao Preto, Brazil; 3 Paulista Center for Diagnosis, Research and Training, Ribeirao Preto, Brazil

## Abstract

**Background:**

Although the motile sperm organelle morphology examination (MSOME) was developed merely as a selection criterion, its application as a method for classifying sperm morphology may represent an improvement in the evaluation of semen quality. The aim of this study was to determine the prognostic value of normal sperm morphology using MSOME with regard to clinical pregnancy (CP) after intrauterine insemination (IUI).

**Methods:**

A total of 156 IUI cycles that were performed in 111 couples were prospectively analysed. Each subject received 75 IU of recombinant FSH every second day from the third day of the cycle. Beginning on the 10th day of the cycle, follicular development was monitored by vaginal ultrasound. When one or two follicles measuring at least 17 mm were observed, recombinant hCG was administered, and IUI was performed 12-14 h and 36-40 h after hCG treatment. Prior to the IUI procedure, sperm samples were analysed by MSOME at 8400× magnification using an inverted microscope that was equipped with DIC/Nomarski differential interference contrast optics. A minimum of 200 motile spermatozoa per semen sample were evaluated, and the percentage of normal spermatozoa in each sample was determined.

**Results:**

Pregnancy occurred in 34 IUI cycles (CP rate per cycle: 21.8%, per patient: 30.6%). Based on the MSOME criteria, a significantly higher percentage of normal spermatozoa was found in the group of men in which the IUI cycles resulted in pregnancy (2.6+/-3.1%) compared to the group that did not achieve pregnancy (1.2+/-1.7%; *P *= 0.019). Logistic regression showed that the percentage of normal cells in the MSOME was a determining factor for the likelihood of clinical pregnancy (OR: 1.28; 95% CI: 1.08 to 1.51; *P *= 0.003). The ROC curve revealed an area under the curve of 0.63 and an optimum cut-off point of 2% of normal sperm morphology. At this cut-off threshold, using the percentage of normal sperm morphology by MSOME to predict pregnancy was 50% sensitive with a 40% positive predictive value and 79% specificity with an 85% negative predictive value. The efficacy of using the percentage of normal sperm morphology by MSOME in predicting pregnancy was 65%.

**Conclusions:**

The present findings support the use of high-magnification microscopy both for selecting spermatozoa and as a routine method for analysing semen before performing IUI.

## Background

Intrauterine insemination (IUI) is a simple and non-invasive procedure that is used to treat couple subfertility. In many cases, IUI allows one to avoid more complex assisted reproduction techniques (ART) such as *in vitro *fertilization (IVF) and intracytoplasmic sperm injection (ICSI). On the other hand, IUI yields varied results as a consequence of the large number of variables that are involved in the treatment, including the aetiology of infertility, the age of the couple, ovarian stimulation protocols, timing and number of inseminations; this has raised doubts regarding the actual effectiveness of IUI [1]. The most common indications for IUI are cervical hostility, mild male factor, mild endometriosis, ovarian dysfunction, and unexplained infertility [2]. In regard to male infertility, there is insufficient information regarding the effectiveness of IUI, and more data are required to either recommend or advise against IUI in these situations [3]. Because one of the most common indications for IUI is mild male factor, and in view of the doubts regarding the efficacy of the procedure with respect to this indication, it would be of high clinical interest to determine the semen parameters that could serve as predictive factors.

Innovative methods for the selection of sperm in ART [4-9] have been published, providing fresh insight into the correlation between sperm quality and clinical results. On the other hand, the value of conventional semen analysis has been the subject of debate. By analysing semen, clinicians expect to obtain a clear indication of the male's fertilisation potential, which is not provided by conventional evaluation (except in particular situations such as total teratozoospermia and globozoospermia) [10,11]. Although none of the semen parameters (or even the functional test)--analysed either separately or jointly--can be considered definitive, morphology has been consistently shown to be the most reliable indicator of male fertility. Diverse studies that originated primarily from IVF/ICSI programmes and IUI corroborate the sensitivity of morphology as a prognostic factor [10-20].

To test the hypothesis that subtle sperm organelle malformations [21,22] are associated with ART results, Bartoov *et al. *[23,24] developed a method of evaluating human spermatozoa in real-time at high magnification; this method is called the motile sperm organelle morphology examination (MSOME). MSOME is performed using an inverted microscope that is equipped with differential interference contrast/Nomarski optics that enable magnification exceeding 6000× [23], which is much higher than the magnification that is typically used by embryologists in selecting spermatozoa for the ICSI procedure (which ranges from 200× to 400×) and is even higher than that which is employed in routine semen examination (1000×). This method favoured the development of intracytoplasmic morphologically selected sperm injection (IMSI), which is based on sperm normality--as defined by the MSOME classification--and is aimed at improving conventional ICSI outcomes by focusing on the existence of a correlation between both sperm morphological abnormalities that can be observed at high magnification and DNA damage [24-28]. Although MSOME was developed merely as a selection criterion, its application as a method for classifying sperm morphology may represent an improvement in the evaluation of semen quality. In the specific case of IUI, seminal evaluation using MSOME could represent an adjunct tool to predict the efficacy of the technique.

To better comprehend the diagnostic/prognostic value of analysing semen morphology using high magnification, this study aimed to evaluate the prognostic value of normal sperm morphology using MSOME classification in predicting clinical pregnancy (CP) after IUI.

## Methods

### Study participants

A total of 156 IUI cycles that were performed in 111 couples at the Center for Human Reproduction Professor Franco Junior were prospectively analysed. The mean ages of the female and male subjects at the time of the IUI cycles were 32.7 ± 3.9 and 35.4 ± 5.6 years, respectively. The indications for IUI included idiopathic infertility in 51.4%, mild male infertility in 23.4%, cervical factor in 15.3%, ovulatory dysfunction in 5.4%, mild endometriosis in 3.6%, and male factor associated with endometriosis in 0.9% of the cases (Table 1). Written informed consent was obtained from all couples on the day of the first IUI cycle. This study received institutional review board approval.

**Table 1:** Clinical and laboratory parameters evaluated according to IUI outcome

Parameters	Total	Pregnancy (n = 34)	No pregnancy (n = 122)	*P*-value
Women's age (years) Mean ± SD	32.8 ± 3.9	32.3 ± 4.0	32.9 ± 3.9	0.45
Range	24-39	25-39	24-39	
Median	33	33	33	
Men's age (years) Mean ± SD	35.5 ± 5.6	34.9 ± 5.0	35.5 ± 5.7	0.59
Range	24-50	27-48	24-50	
Median	34	35	34	
Duration of infertility (years) Mean ± SD	2.8 ± 1.9	2.4 ± 1.9	2.9 ± 1.9	0.25
Range	1-13	1-10	1-13	
Median	2	2	2	
MSOME (%)				
-Normal spermatozoa Mean ± SD	1.5 ± 2.2	2.6 ± 3.1	1.2 ± 1.2	0.019
Range	0-13	0-13	0-13	
Median	1	1.5	0.5	
Follicles ≥17 mm (n) Mean ± SD	1.2 ± 0.4	1.2 ± 0.4	1.2 ± 0.4	0.64
Range	1-2	1-2	1-2	
Median	1	1	1	
Aetiology				
-Idiopathic	51.4%(57/111)	46.2%(12/26)	52.9%(45/85)	0.66
-Male (mild)	23.4%(26/111)	30.8%(8/26)	21.2%(18/85)	
-Cervical	15.3%(17/111)	19.2%(5/26)	14.1%(12/85)	
-Ovulatory	5.4%(6/111)	3.8%(1/26)	5.9%(5/85)	
-Endometriosis	3.6%(4/111)	0	4.7%(4/85)	
-Male (mild) + Endometriosis	0.9%(1/111)	0	1.2%(1/85)	
DNA fragmentation (%) Mean ± SD	16.4 ± 8.0	15.0 ± 7.4	16.7 ± 8.1	0.26
Range	3-37.5	5-35.5	3-37.5	
Median	15	12	15	
Total sperm count (x10^6^/ml) Mean ± SD	79.2 ± 54.4	83.6 ± 43.4	78 ± 57.2	0.24
Range	5-280	15-160	5-280	
Median	68.5	86	62	
Motility (% spermatozoa) (rapid + slow progression) Mean ± SD	63.2 ± 15.4	59.7 ± 14.9	64.2 ± 14.5	0.12
Range	19-93	19-87	19-93	
Median	63	60	66	
Leukocytes (x10^6^) Mean ± SD	0.26 ± 0.26	0.30 ± 0.28	0.25 ± 0.26	0.34
Range	0-1.6	0-1.3	0-1.6	
Median	0.2	0.2	0.2	

### IUI procedures

For ovarian stimulation, each female subject received 75 IU of recombinant FSH (r-FSH; Gonal F, Serono, Brazil) every second day from the third day of the cycle. Beginning on the 10th day of the cycle, follicular development was monitored by vaginal ultrasound, and the dose of r-FSH was changed based on the ovarian response. When one or (at most) two follicles measuring at least 17 mm were observed, recombinant hCG (r-hCG; Ovidrel, Serono, Brazil) was administered.

Each patient underwent two consecutive IUI procedures (12-14 h and 36-40 h after r-hCG). Semen samples were collected into sterile containers by masturbation and immediately used for the IUI procedures. Liquefied fresh semen samples were prepared with an Isolate discontinuous concentration gradient (Irvine Scientific, Santa Ana, CA, USA). The final pellet was resuspended in 0.5 and 0.3 ml modified HTF medium (Irvine Scientific) that was supplemented with 10% human serum albumin (Irvine Scientific) for the first (12-14 h) and second (36-40 h) inseminations.

All of the IUI procedures were performed using a Frydman catheter (Frydman Classic Catheter 4.5 CCD Laboratoire CCD; Paris, France) guided by abdominal ultrasound using a 3.5 MHz convex transducer (Aloka SSD-1100; Aloka Co. Ltd., Tokyo, Japan) by the same physician. Patients with a full bladder were placed in the lithotomy position, and the cervix was exposed using a bivalve speculum. For all of the IUI procedures, the catheter passed smoothly through the cervix (without the need for uterine fixation clamps) with clear visualisation of the catheter tip upon ultrasound. In each insemination, the medium containing the spermatozoa was gently expelled into the uterine cavity under ultrasound monitoring. Following IUI, the catheter was immediately and carefully removed, and the patient was allowed to rest in bed for 15 min. Each patient received luteal phase supplementation with vaginal natural progesterone.

Pregnancy was diagnosed based on an increase in serum ß-hCG concentration 14 days after IUI. Clinical pregnancy was defined as the presence of a gestational sac that was accompanied by an image of the embryo/fetal cardiac activity on transvaginal ultrasound 4 weeks after IUI.

### Determination of sperm morphology by MSOME

Before the IUI procedure, semen samples were analysed for standard semen quality parameters according to the World Health Organization [29] using MSOME and for DNA fragmentation using the TUNEL assay as previous described [30,31]).

For MSOME, the liquefied fresh semen samples were prepared using the Isolate discontinuous concentration gradient (Irvine Scientific). The final pellet was resuspended in 0.2 ml modified human tubal fluid (HTF) medium (Irvine Scientific). A 1-μl aliquot of the sperm cell suspension was transferred to a 5-μl microdroplet of modified HTF medium containing 7% polyvinylpyrrolidone solution (PVP medium; Irvine Scientific). This microdroplet was placed in a sterile glass dish (FluoroDish; World Precision Instruments, USA) under sterile paraffin oil (Ovoil-100; VitroLife, Göteborg, Sweden). The sperm cells that were suspended in the microdroplet were placed on a microscope stage above an Uplan Apo ×100 oil/1.35 objective lens that was previously covered with a droplet of immersion oil. In this manner, suspended motile sperm cells in the observation droplet were then examined under high magnification using an inverted microscope (Eclipse TE 2000U; Nikon, Japan) that equipped with high-power differential interference contrast optics (DIC/Nomarski). The images were captured using a colour video camera containing effective picture elements (pixels) for high-quality image production and visualised on a colour video monitor. The morphological evaluation was performed on a monitor screen, and the combined calculated magnification was 8450× (total magnification: objective magnification = 100 ×; magnification selector = 1.0 ×; video coupler magnification = 1.0 ×; calculated video magnification = 84.50).

A spermatozoon was classified as morphologically normal when it exhibited a normal nucleus, acrosome, postacrosomal lamina, neck and tail and did not present a cytoplasm around the head [23]. The subcellular organelles were morphologically classified as follows on the basis of the presence of specific malformations that were defined according to the arbitrary descriptive approach that was reported by Bartoov et al. [23] using studies utilising transmission and scanning electron microscopy: an absent, partial or vesiculated acrosome; an absent or vesiculated post-acrosomal lamina; a neck that was abaxial, disordered or showing a cytoplasmic droplet; an absent, coiled, broken, multiple or short tail.

**Figure 1:**
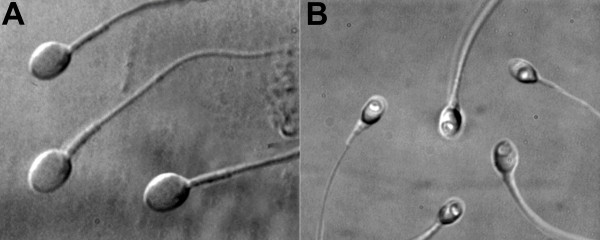
**Sperm morphology**. **A: **Normal spermatozoa observed at high magnification (8400×); **B: **Spermatozoa with large nuclear vacuoles observed at high magnification (8400×).

With respect to the nucleus and according to transmission electron microscopy estimates [23,32], the normal morphological state was defined by the shape and content of the chromatin. The criterion for normality of nuclear shape was a smooth, symmetric and oval configuration. Normal means for length and width were estimated as 4.75 ± 2.8 and 3.28 ± 0.20 μm, [23], respectively, wherein the shape was classified as abnormal when measuring more than 2 SD on at least one of the axes (length: ≥5.31 or ≤4.19 μm; width: > 3.7 or < 2.9 μm). For a rapid evaluation of nuclear shape, a fixed, transparent, celluloid form of sperm nucleus fitting the criteria was superimposed on the examined cell (chablon construction based on ASTM E 1951-2 [33]). The criterion for normal chromatin content was the absence of vacuoles occupying > 4% of the sperm nuclear area. Figure 1A shows normal spermatozoa as analysed by MSOME.

The same technician performed all of the sperm selections. At least 200 motile spermatozoa per sample were evaluated, and the percentages of normal spermatozoa were determined. The analysis lasted 30-60 min/sample.

### Statistical analysis

Data management and univariate analysis were performed using the StatsDirect statistical software (Cheshire, UK) to compare the variables between the group of cycles in which the IUI procedure resulted in pregnancy and the group in which the IUI procedure did not result in pregnancy. The analysis was performed at the cycle level (i.e., each cycle was considered as a separate unit for analysis). To compare the means of the continuous variables, the non-parametric Mann-Whitney test was used if the continuous variables were not normally distributed, and the Student's *t*-test was used if the continuous variables were normally distributed. The results are expressed as the arithmetic mean ± standard deviation (SD), range and median. For categorical variables, the chi-square test was used to check their association between groups, and the results are expressed as a percentage. Univariate logistic regression was also used to estimate the value of an independent variable in predicting the likelihood of becoming pregnant in an IUI cycle. The odds ratio (OR) and 95% confidence interval (CI) constituted the descriptive analysis.

Receiver operating characteristic curves (ROC) were constructed to examine the performance of the significant variables as identified by the previous statistical tests (i.e., to evaluate the ability of a variable to predict pregnancy after an IUI cycle). An optimised threshold was determined. The discriminative performance of the model was assessed by the area under the ROC curve. Sensitivity was defined as the fraction of cycles that resulted in a pregnancy that was predicted correctly, and specificity was defined as the fraction of cycles not resulting in a pregnancy that was predicted correctly. StatsDirect requires the following two columns of data for each ROC plot: one with test results in cases where the condition being tested is known to be present (pregnancy positive) and the other with test results in known negative (pregnancy negative) cases. Sensitivity is then plotted against (1-specificity). StatsDirect calculates directly the area under the ROC curve using an extended trapezoidal rule [34] and by a non-parametric method that is analogous to the Wilcoxon/Mann-Whitney test [35]. A confidence interval was constructed using DeLong's variance estimate [36].

## Results

Pregnancy occurred in 34 IUI cycles (CP rate per cycle: 21.8%, per patient: 30.6%). A significantly higher incidence of normal spermatozoa according to the MSOME criterion was found in the group of men in which the IUI cycles resulted in pregnancy (2.6 ± 3.1%) compared to the group that did not achieve pregnancy (1.2 ± 1.7%; *P *= 0.019). The laboratory and clinical parameters that were evaluated according to IUI outcome are shown in Table 1. With the exception of the percentage of normal spermatozoa, there were no significant differences between the groups.

Logistic regression revealed that the percentage of normal cells in the MSOME analysis was a determinant of the likelihood of achieving clinical pregnancy (OR: 1.28; 95% CI: 1.08 to 1.51; *P *= 0.003). On the other hand, logistic regression did not reveal a statistically significant (*P *> 0.05) association between IUI outcome (CP) and the other parameters that were analysed, including the woman's age (OR: 0.96; 95% CI: 0.88 to 1.06) and man's age (OR: 0.98; 95% CI: 0.91 to 1.05), the duration of infertility (OR: 0.81; 95% CI: 0.63 to 1.03), DNA fragmentation (OR: 0.97; 95% CI: 0.97 to 1.02), the number of follicles ≥17 mm (OR: 1.40; 95% CI: 0.53 to 5.21), and other semen characteristics, including total sperm count (OR: 1.00; 95% CI: 0.99 to 1.00), progression motility (OR:0.98; 95% CI: 0.95 to 1.00), and leukocytes (OR:1.77; 95% CI: 0.46 to 6.70). Table 2 summarises these results.

**Table 2:** Univariate analysis of pregnancy occurrence after intrauterine insemination by logistic regression

Parameter	Logistic Regression		
	Odds Ratio	95% CI	*P*-value
Women's age (years)	0.96	0.87 to 1.06	0.44
Men's age (years)	0.98	0.91 to 1.05	0.61
Duration of infertility (years)	0.81	0.63 to 1.03	0.10
MSOME (%) -Normal spermatozoa	1.28	1.08 to 1.51	0.003
Follicles ≥17 mm (n)	1.40	0.53 to 3.68	0.49
DNA fragmentation (%)	0.97	0.97 to 1.02	0.26
Total sperm count (x10^6^/ml)	1.00	0.99 to 1.01	0.59
Motility (% spermatozoa)(rapid + slow progression)	0.98	0.96 to 1.01	0.13
Leukocytes (x10^6^) (mean ± SD)	1.77	0.46 to 6.70	0.39

CI: confidence interval

**Figure 2:**
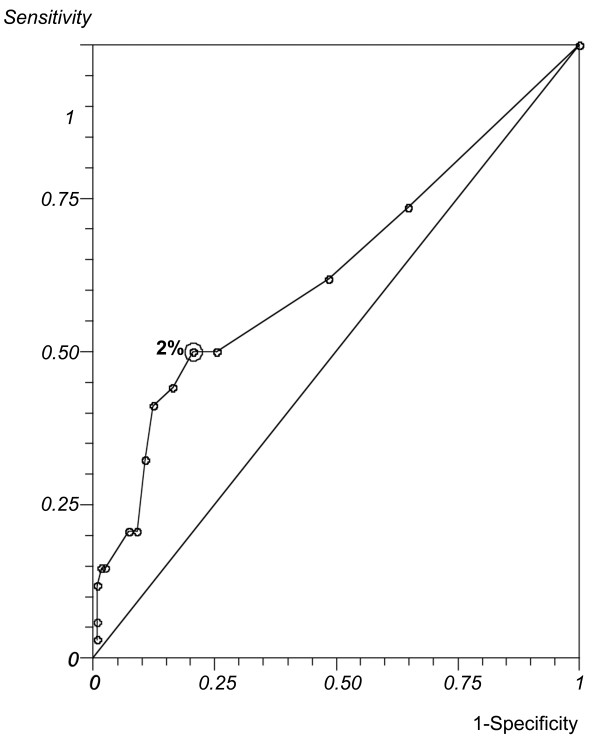
**ROC Curve**. ROC curve analysis for the percentage of normal sperm morphology using MSOME as a prognostic factor regarding clinical pregnancy after intrauterine insemination. The area under the curve is 0.63. The best discriminating percentage (2%) is indicated. At this cut-off level, the ability of the percentage of sperm normal form by MSOME to predict pregnancy showed 50% sensitivity with a 40% positive predictive value and 79% specificity with an 85% negative predictive value. The efficacy was 65%.

The ROC curve was created only for the percentage of normal sperm morphology s by MSOME (which was the only variable that differed significantly between the groups in the previous analysis). The ROC curve (Figure 2) had an area under the curve of 0.63 (95% CI: 0.52-0.78) (see Methods), indicating that the percentage of normal forms as analysed by MSOME had reasonable prognostic potency for predicting pregnancy after IUI. Setting the threshold at 2% offered the optimal compromise between specificity (50%) and sensitivity (79%) and between positive predictive value (40%) and negative predictive value (85%). At this cut-off level, the efficacy of the percentage of normal sperm morphology by MSOME in predicting pregnancy was 65%.

## Discussion

The rationale for the IUI procedure is to maximise the number of motile spermatozoa at the site of fertilisation to maximise the likelihood of achieving pregnancy. Although IUI is used widely in the treatment of couple subfertility, its true efficacy is yet to be determined, particularly when the technique is employed due to male subfertility [1]. According to Merviel *et al. *[37], the most important predictive factors for pregnancy in IUI cycles are the recruitment of two preovulatory follicles that are > 16 mm in a woman of age ≤30 years and a concentration ≥5 million motile spermatozoa and teratospermia ≤70% after the preparation of semen by a discontinuous concentration gradient. Another study reported that IUI that was performed for male factor subfertility has a low possibility of success when the woman's age is > 35 years, the number of motile spermatozoa that are inseminated is < 5 million, or normal sperm morphology--as defined by the criteria of the World Health Organization--is < 30% [38].

On the other hand, Dorjpurev *et al. *[39] showed that with a motility rate of ≥30% and a motile sperm concentration of ≥10 million/ml, IUI can be a useful tool for treating male subfertility. Kamath *et al. *[40] reported that a duration of infertility of less than 5 years and a total motile spermatozoa count of more than 10 million are related to a better prognosis in IUI, and they found a trend toward a higher pregnancy rate with endometrial thickness > 6 mm. Wainer *et al. *[41] reported that a minimum of 5 million motile spermatozoa should be inseminated only when the normal morphology of the sperm after preparation is < 30%, as the quantity compensates--at least in part--for poor seminal quality; they further noted that if these parameters cannot be reached, *in vitro *fertilization should be recommended. Other clinical parameters have also been evaluated, including the comparison of recombinant FSH and highly purified FSH for ovarian stimulation [42], the use of GnRH antagonists to avoid a premature LH surge [43,44], the association of chromatin condensation in spermatozoa with conventional semen parameters [45], and the use of a soft versus firm catheter for the insemination procedure [46], all of which were designed to identify predictive factors of the likelihood of achieving pregnancy in IUI.

Unfortunately, MSOME is not typically applied beyond its use in sperm selection for the ICSI procedure. In fact, to the best of our knowledge, the present study is the first to analyse MSOME as a prognostic factor for IUI; thus, our results cannot be compared with other results. However, our data are in agreement with other studies that used other morphological sperm evaluation criteria. In fact, morphological sperm alterations seem to confer a significantly poorer outcome in IUI [37,47,48] and are reported to be a more relevant semen parameters than sperm concentration and sperm motility [49]. Karabinus and Gelety [50] stated that pregnancy rates per cycle after IUI were not different when the percentage of morphologically normal sperm (strict criteria) in raw semen was 5%, 5-9%, 10-19%, 20-29%, or ≥30% (with rates of 6.55 ± 3.9,13.6 ± 3.2, 8.8 ± 2.4, 7.1 ± 2.5, and 9.7 ± 3.3%, respectively). Thus, the authors concluded that IUI appears to be a successful treatment modality for male factor infertility even in cases in which the percentage of morphologically normal sperm in raw semen is quite low.

On the other hand, the morphological criteria for assessing spermatozoa quality according to parameters of the World Health Organization were recently modified (with a normal morphology cut-off value of > 4% in the 2010 WHO manual) [51], demonstrating that there is still a controversy regarding the definition of a morphologically normal spermatozoon. It would be of great clinical value to establish a laboratory parameter that could reliably and more efficiently predict the outcome of IUI to both improve the outcome and optimise the indications of the technique. Recently, our group suggested that MSOME should be included among the routine criteria for semen analysis, given that it is a much stricter criterion of sperm morphology classification than the Tygerberg criterion [27]. In addition, we also demonstrated that MSOME appears reliable for analysing semen [52].

One of the most important alterations that can be observed with MSOME is the presence of large nuclear vacuoles (LNV; see Figure 1B). We consider LNV as those that occupy more than 50% of the nuclear area [52]. LNV are specific sperm alterations that can be observed at high magnification, and their presence has been shown to have clinical implications. Based on electron microscopy data, Bartoov et al. [23,53] and Berkovitz et al. [32] assumed that nuclear vacuoles indicate a chromatin abnormality. Other studies confirmed the association between nuclear vacuoles at high magnification and chromatin damage. Berkovitz et al. [54] graded the severity of nuclear morphological alterations, highlighting principally the presence of large vacuoles and suggesting that vacuolisation of the sperm nucleus reflects some underlying chromosomal or DNA defect. Franco et al. [31,55] demonstrated an association between large nuclear vacuoles and the presence of DNA fragmentation, denaturation and protamination in the spermatozoa. Garolla et al. [56] showed that the presence of nuclear vacuoles affects mitochondrial function, chromatin status, and aneuploidy rate. Using electron microscopy, Toshimori and Ito [57] associated the presence and content of nuclear vacuoles with DNA damage. In addition, the authors emphasised that IMSI/MSOME aids in identifying vacuoles. Oliveira et al. [28] observed a significant positive correlation between the percentage of sperm that contain large nuclear vacuoles and the percentage of DNA fragmentation. Gopalkrishnan *et al. *[58] found that the chromatin material of spermatozoa from men whose partners presented with early pregnancy loss was often found to be either compact or partially compact with irregular nuclear borders and large vacuoles. Moreover, a recent study has related LNV to the absence of acrosome reaction, which could have deleterious effects on the processes of fertilization and embryo development [59]. On the other hand, it has been clearly established that the capacity of human sperm to fertilise an oocyte and produce an embryo with a high potential for implantation and development depends on the sperm cell's DNA integrity [56]; thus, MSOME could provide important information regarding the likelihood of success with ART.

Many investigators have reported the outcome of *in vitro *fertilization techniques according to MSOME [24,25,32,52,60-63], but this evaluation has not been employed previously to determine the success rate of IUI. The present study investigated the outcome of IUI compared with semen morphology as assessed with MSOME. We observed a significantly higher pregnancy rate with a higher percentage of normal cells according to the MSOME parameters. However, a logistic regression analysis failed to identify a significant correlation between treatment outcome and other characteristics such as patient age, duration of infertility, the number of follicles and other semen parameters. This is in contrast with other published reports [1,37,39]. The lack of consensus in the literature clearly highlights the difficulties in finding reliable prognostic parameters for determining IUI outcome.

With regard to the percentage of morphologically normal spermatozoa, we observed an optimum cut-off point of 2%. With this cut-off point, the efficacy of the exam in predicting pregnancy following IUI reached 65%, as shown by the ROC curve. Because there are doubts regarding the efficacy of IUI for male factor, MSOME could also play a role as a method for the classification of sperm morphology to offer an additional parameter for the indication of the technique. Besides the woman's age, the duration of infertility and the concentration of motile spermatozoa, which are predictive factors that are considered to be important by other investigators, our data suggest that 2% morphologically normal spermatozoa at MSOME is desirable to maximise the likelihood of pregnancy following IUI.

The accuracy with which the morphological normality of spermatozoa can be assessed depends on the resolution power of the optical magnification system. Spermatozoa that appear to be morphologically normal at 1000× magnification may in fact have various structural abnormalities that can only be detected at higher optical magnification (> 6000×). Few studies have attempted to analyse MSOME as a morphological classification method for semen. Using MSOME, Bartoov *et al. *[23] reported an incidence of sperm normality of 2.9 ± 0.5% (range 0-5%). In our study, we found an incidence of 1.5 ± 2.2% (0-13%). Differences with regard to the observation of nuclear vacuoles can explain the divergence found in the normality rates between MSOME and other morphological criteria. Employing the Tygerberg criteria, Bar-Chama *et al. *[64] analysed the number of sperm vacuoles in a series of 1,295 fresh post-processed sperm samples. They found vacuoles in only 19.5% (253) of the samples. On the other hand, MSOME revealed that the ejaculates of males who were routinely referred for ICSI exhibit an average of 30-40% of spermatozoa with vacuolated nuclei [54]. In addition, there is divergence in the quantification (i.e., normal or abnormal) of the presence of nuclear vacuoles. Using MSOME, the nuclear chromatin content is considered to be abnormal if it contained one or more vacuoles that occupied more than 4% of the nuclear area [23]. Nevertheless, other criteria (for example, the Tygerberg criteria) are much more tolerant with regard to the presence of vacuoles. A head is considered to be defective only when > 20% of its area is occupied by unstained vacuolar areas [29,65].

On the other hand, Bartoov *et al. *[23] emphasised that whereas a routine morphological examination is applied to semen samples as a whole, MSOME focuses only on the fraction of motile spermatozoa. Analysing only the motile spermatozoa by MSOME can confer an additional advantage, as it will provide information regarding the sample fraction with a higher potential for achieving fertilization and development. Even though analyses using other criteria also employ high magnification, the characteristics of these procedures (i.e., fixation and staining) preclude the possibility of obtaining information for the motile portion.

In conclusion, the present study suggests that the use of MSOME for semen evaluation can be a reliable predictor of the incidence of normal forms in a sperm sample. Performing MSOME prior to the IUI procedure can represent a valuable tool to support or contraindicate this relatively inexpensive treatment and ultimately contribute to the indication of more complex ART procedures, thereby avoiding a loss of time. The present findings support the use of high-magnification microscopy both for selecting spermatozoa and as a routine method for semen analysis with potential clinical applications.

## Competing interests

The authors declare that they have no competing interests.

## Authors' contributions

LDA was responsible for designing and coordinating the study. All of the authors were responsible for the collection, analysis, and interpretation of the data presented in the manuscript. JBAO, RLRB, MC and JGF were responsible for the statistical analyses and for writing the manuscript. JBAO and JGF were responsible for revising the manuscript. All of the authors have read and approved the final manuscript.
